# Surface Localization of Glucosylceramide during *Cryptococcus neoformans* Infection Allows Targeting as a Potential Antifungal

**DOI:** 10.1371/journal.pone.0015572

**Published:** 2011-01-21

**Authors:** Ryan Rhome, Arpita Singh, Talar Kechichian, Monica Drago, Giulia Morace, Chiara Luberto, Maurizio Del Poeta

**Affiliations:** 1 Departments of Biochemistry and Molecular Biology, Medical University of South Carolina, Charleston, South Carolina, United States of America; 2 Departments of Microbiology and Immunology, Medical University of South Carolina, Charleston, South Carolina, United States of America; 3 Department of Craniofacial Biology, Medical University of South Carolina, Charleston, South Carolina, United States of America; 4 Division of Infectious Diseases, Medical University of South Carolina, Charleston, South Carolina, United States of America; 5 Dipartimento di Sanita' Pubblica, Microbiologia-Virologia, Universita' degli Studi di Milano, Milan, Italy; Institut Pasteur, France

## Abstract

*Cryptococcus neoformans* (*Cn*) is a significant human pathogen that, despite current treatments, continues to have a high morbidity rate especially in sub-Saharan Africa. The need for more tolerable and specific therapies has been clearly shown. In the search for novel drug targets, the gene for glucosylceramide synthase (*GCS1*) was deleted in *Cn,* resulting in a strain (*Δgcs1*) that does not produce glucosylceramide (GlcCer) and is avirulent in mouse models of infection. To understand the biology behind the connection between virulence and GlcCer, the production and localization of GlcCer must be characterized in conditions that are prohibitive to the growth of *Δgcs1* (neutral pH and high CO_2_). These prohibitive conditions are physiologically similar to those found in the extracellular spaces of the lung during infection. Here, using immunofluorescence, we have shown that GlcCer localization to the cell surface is significantly increased during growth in these conditions and during infection. We further seek to exploit this localization by treatment with Cerezyme (Cz), a recombinant enzyme that metabolizes GlcCer, as a potential treatment for *Cn.* Cz treatment was found to reduce the amount of GlcCer *in vitro*, in cultures, and in *Cn* cells inhabiting the mouse lung. Treatment with Cz induced a membrane integrity defect in wild type *Cn* cells similar to *Δgcs1*. Cz treatment also reduced the *in vitro* growth of *Cn* in a dose and condition dependent manner. Finally, Cz treatment was shown to have a protective effect on survival in mice infected with *Cn.* Taken together, these studies have established the legitimacy of targeting the GlcCer and other related sphingolipid systems in the development of novel therapeutics.

## Introduction

*Cryptococcus neoformans* (*Cn*) is one of the major fungal human pathogens and continues to have clinical significance despite the development of antifungal drugs. Typically, *Cn* is an opportunistic pathogen causing a significant disease in immunocompromised patients [1], and it has been shown to cause serious pulmonary infections in individuals with fully functioning immune systems [2]. The initial portal of infection in humans is through the lung, when spores or desiccated yeast cells are inhaled from environmental reservoirs such as pigeon droppings [2]. Within the lung, *Cn* acts as a facultative intracellular pathogen, growing in either the extracellular spaces of the alveoli or intracellularly in the acidic phagolysosome of the alveolar macrophages. In some patients, this infection progresses, disseminating to the bloodstream where it can cause infections in most major organ systems. The most clinically important aspect of this process is when *Cn* enters the central nervous system (CNS), where it thrives, becoming the most common cause of fungal meningoencephalitis in the world. The Center of Disease Control and Prevention (CDC) estimates that over 1 million new cases/year of cryptococcosis are reported worldwide in patients with acquired immune deficiency syndrome (AIDS), with over half those affected dying of the infection, making deaths caused by cryptococcosis in patients with HIV in sub-Saharan Africa more frequent than deaths caused by tuberculosis [3], [4]. This is drastic increase considering that prior to the mid-1950s, fewer than 300 cases of cryptococcosis had been reported in the medical literature (reviewed in [5]). Thus, studies looking at new treatment strategies are needed.

Understanding the pathophysiology of *Cn* is crucial to the development of proper treatments. Current clinical standard for *Cn* involves amphotericin B plus 5-fluorocytosine but problems with tolerance of their side effect combined with the existence of resistant strains has led to an ongoing search for more tolerable and efficacious drug treatments.

The general characteristics of an ideal drug target for a pathogen would be one that targets the biology of the microbe with little to no effect on the host. For these reasons, one growing field of study in clinically related microbiology is the sphingolipid pathways of the organism of interest. Fungal sphingolipid pathways are distinct in many ways from their mammalian analogs, both in the enzymes and products involved. The sphingolipid biosynthetic pathway has been implicated in the growth and virulence of several clinically significant fungi (reviewed in [6], [7], [8], [9]), the best studied of these being *Cn* [10], [11], [12], [13], [14], [15], [16].

Of note, glucosylceramide synthase (Gcs1) has been implicated in the growth cycle of *Aspergillus* [17] and directly linked to virulence in *Cn* [18]. The deletion of the gene encoding this enzyme in *Cn* results in a strain (*Δgcs1*) that does not cause morbidity or mortality in inhalation mouse models of cryptococcosis. Instead, these cells are contained within granulomatous structures in the lung. Interestingly, the same strain (*Δgcs1*) shows comparable mortality to wild type when introduced intravenously. This suggests that GlcCer and the ability to grow extracellularly are crucial to virulence *early* in the process of infection and dissemination. Further investigation of this phenotype reveals that *Δgcs1* has *in vitro* deficits in growth at conditions found in the extracellular spaces of the lung (5% CO_2_ and pH 7.4). *Δgcs1* therefore has growth characteristics similar to an obligate intracellular pathogen, because it is still able to grow at the low pH (4.0) found in the macrophage's phagolysosome.

The various functions of glucosylceramide (GlcCer) in several fungi and plants are still being studied [19], [20]. The presence of GlcCer in fungi has been associated with the ability to grow at more alkaline pH [21]. GlcCer is known to localize to the cell surface in *Cn*, particularly at sites where daughter cells are budding from the mother [22]. GlcCer also is found in *Cn* in vesicles secreted through the cell wall to the extracellular space [23], [24] that contain polysaccharides used in the synthesis of the capsule. Patients with cryptococcosis elicit an antibody response against glucosylceramide [25]. Some studies have suggested that GlcCer may be a good target for antifungal therapies. Antibodies isolated from these patients have both shown to inhibit the growth of *Cn in vitro* [22]. Using anti-GlcCer antibodies in passive immunization of mice infected with *Cn* showed a protective effect [26]. Taken together, these studies suggest that Gcs1 and/or its product, GlcCer, would be ideal targets for potential anti-cryptococcal therapies. To date, no specific inhibitors for fungal Gcs1 exist. Inhibitors of the mammalian homolog have no significant effect on the cryptococcal enzyme (unpublished Del Poeta data).

The purpose of this work is to characterize the role of GlcCer in the conditions where the *Δgcs1* strain fails to thrive and to evaluate the potential of targeting GlcCer pharmacologically as an anti-cryptococcal therapy. Human patients with Gaucher's disease have genetic defects in the catabolism of GlcCer. As a therapy for this disease, a human recombinant GlcCer glucosidase called Cerezyme (Cz) is given exogenously. This enzyme hydrolyzes the glucose moiety from the first carbon position of the GlcCer molecule, yielding glucose and ceramide. Here we report the investigation of the behavior of GlcCer in *Cn* biology during infection and the exploitation of this glycosphingolipid as a potential therapy for *Cn* infection.

## Results

### Production of IgM monoclonal antibodies against *Cn* GlcCer

**Figure 1:**
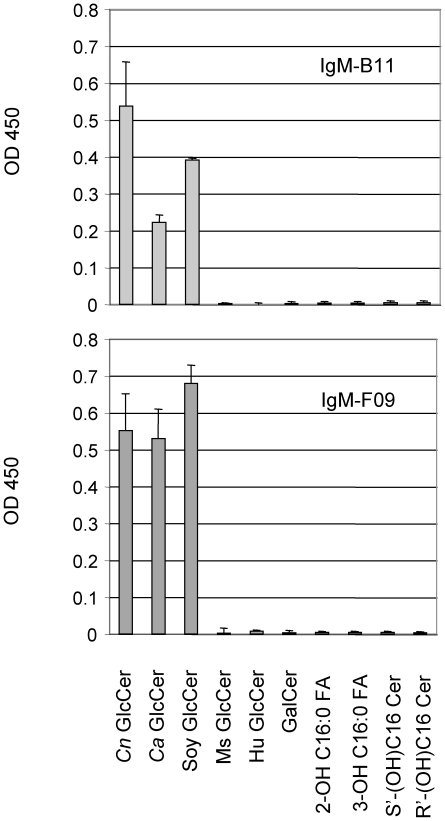
Specificity of IgM anti-GlcCer monoclonal antibody. Monoclonal IgM-B11 and F09 antibody recognize *Cryptococcus neoformans (Cn)*-, *Candida albicans (Ca)*- and soy-GlcCer but not mouse (Ms)-GlcCer, human (Hu) GlcCer, galactosylceramide (GalCer), 2-hydroxy-C16:0 fatty acid, 3-hydroxy-C16:0 fatty acid, S'-hydroxy-C16 ceramide (Cer) or R'-hydroxy-C16 Cer. Approximately, 5 µg of each lipid were used in the assay.

Using the hybridoma technique described in the Materials and Methods, several IgM clones were obtained and two clones (IgM-B11 and IgM-F09) were chosen for further studies. IgM-B11 contains a kappa light chain whereas IgM-F09 contains a lambda light chain. Using ELISAs, illustrated in the Figure 1, both IgM-B11 and IgM-F09 antibodies reacted with GlcCer isolated from *Cn*, *Ca,* and soy but not with human GlcCer. Neither IgM-F09 nor IgM-B11 reacted with mouse GlcCer. To make sure that the antibodies are specifically directed to GlcCer and not to the ceramide back bone, we tested whether these antibodies would react with different species of ceramides, fatty acids, or with a ceramide harboring a different sugar moiety, such as galactosylceramide (GalCer) and found no interaction (Figure 1), confirming that the β-glucose unit is essential for the binding of both IgM-B11 and IgM-F09 antibody to GlcCer. Thus, these antibodies were used for the studies indicated below.

### GlcCer localizes to the cell surface of *Cn* during intranasal infection

**Figure 2:**
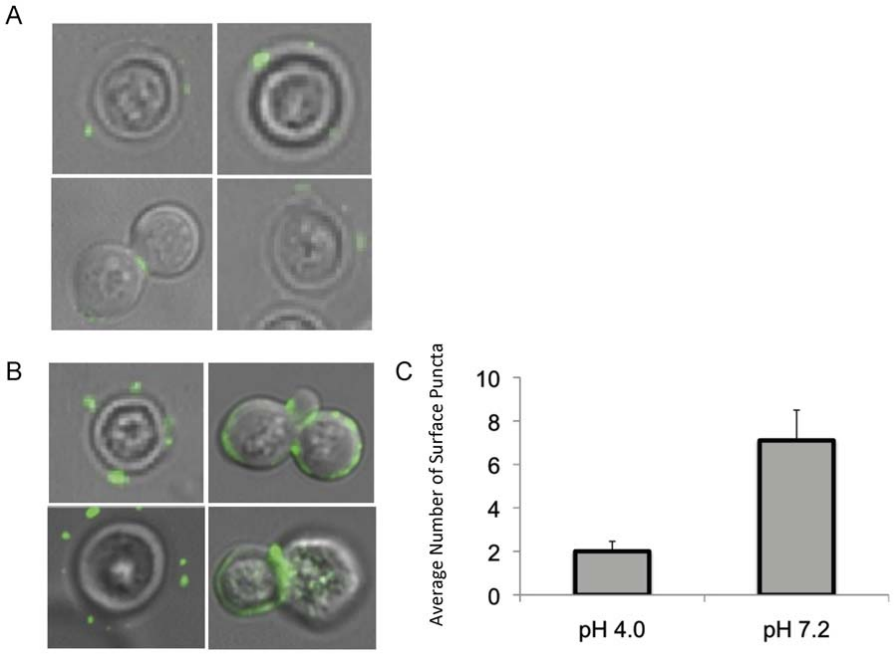
Localization of GlcCer in *Cn* grown *in vitro* at high CO_2_ and either acidic or neutral pH. Indirect immunofluorescence was used to determine the localization of GlcCer in wild type *Cn* in media of either acidic (A) or neutral (B) pH. Primary antibody used is an anti-Cn GlcCer monoclonal antibody developed by our lab. The secondary was an isotype-specific FITC-conjugated antibody, and confocal microscopy was used to analyze the images. The amount of surface puncta per cell was quantified by counting puncta from three large fields of cells, averaged by cell number (C).

**Figure 3:**
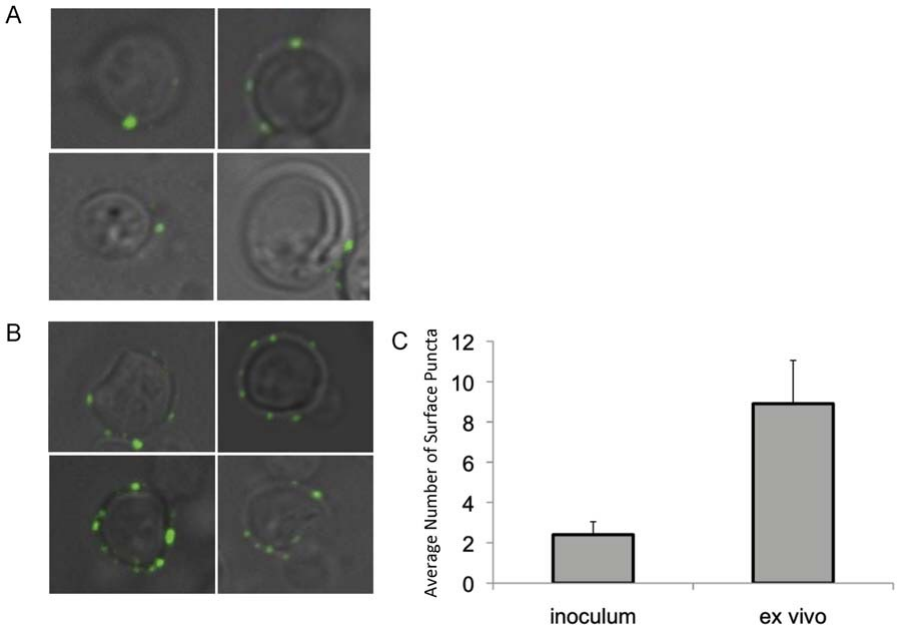
Localization of GlcCer in *Cn* during infection. Wild type mice were infected with 5×10^7^ cells of H99 and allowed to incubate for 48 hours. The cells were then removed from the lungs of the mice by broncheoalveolar lavage (BAL). Indirect immunofluorescence was used to determine the localization of GlcCer in wild type *Cn* in the inoculum used to infect (A) as compared to the cells recovered from the BAL (B). The amount of surface puncta per cell was quantified by counting puncta from three large fields of cells, averaged by cell number (C).

Previous studies have shown that GlcCer localizes at the surface of the cell during infection. Additionally, Δ*gcs1* strain lacking GlcCer shows impaired ability to grow on media supplemented with sodium dodecyl sulfate (SDS), a detergent that affects fungal membranes. Suggesting a possible role of GlcCer in membrane stability, we examined the localization of GlcCer at conditions that are restrictive or permissive to growth of the Δ*gcs1* strain. Using indirect immunofluorescence with the IgM F09 monoclonal anti-*Cn* GlcCer antibody as a primary produced in our laboratory (please see Materials and Methods). GlcCer signals were assessed using cells grown overnight at high CO_2_ in minimal media in either pH 4.0 (Figure 2A) or 7.2 (Figure 2B). These are presented here as merged fluorescence and light microscope images. The number of surface *puncta* per cell were counted in three separate fields of at least 75 cells total (Figure 2C) and averaged from three separate experiments. The *Cn* cells grown in minimal media at pH 7.2 showed a significant increase in surface *puncta* per cell compared to those grown in pH 4.0. These growth conditions mimic those seen in the extracellular spaces of the lung during infection, and therefore we hypothesize that this increased surface localization would be seen in cells taken from infection models. To confirm this, mice were infected with *Cn* for 48 hours, and the cells were removed by BAL procedure. These cells were subjected to the same indirect immunofluorescence protocol as above (Figure 3A and 3B) and surface *puncta* were quantified (Figure 3C). The cells taken *ex vivo* from murine infection models showed a similar pattern of GlcCer surface localization as those grown in the *in vitro* conditions used to mimic the physiological conditions seen in extracellular space of the lung. In all these experiments, negative controls with the Δ*gcs1* strain and with WT *Cn* incubated with only secondary antibody were used, and showed no appreciable immunofluorescence signal (data not shown).

**Figure 4:**
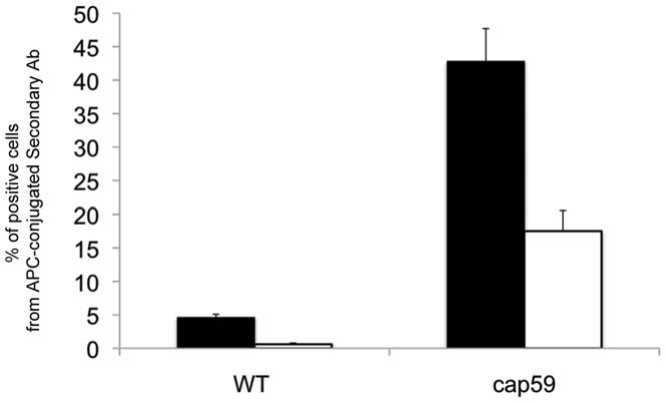
Amount of cells with GlcCer available on the cell surface of *Cn*. The amount of GlcCer available on the surface of the *Cn* was assessed in wild type and the acapsular Δcap59 strain using flow cytometry. The cells were grown at either pH 7.2 (black bars) or 4.0 (white bars). These graphs represent the percentage of cells with positive signal from APC-conjugated secondary antibodies bound to anti-fungal GlcCer primary antibodies on the cell surface. There was negligible signal from the *Δgcs1* strain (negative control, subtracted as background).

One potential confounding variable in this scenario is the capsule size. To determine if this same differential localization occurs in an acapsular strain, *Δcap59*, flow cytometry was used, using the monoclonal IgM F09 anti-*Cn* GlcCer antibody as the primary (Figure 4). Even in the acapsular strain, the amount of GlcCer available on the surface for flow cytometry was increased significantly in the strain when grown at pH 7.2 as compared to growth at pH 4.0. This suggests that the localization observed by indirect immunofluorescence is not due to differences in capsule size during growth in these conditions. Immunofluorescence protocols with *Δcap59* were attempted, however separation of the cells from each other after fixation was difficult and low-yield. Using the flow cytometry protocol, separation was greatly improved and clumped cells could be excluded from analysis.

### Increased GlcCer on cell surface is not a result of increased expression, enzyme activity, production, or reduced degradation in conditions seen in the lung during infection

The increased localization of GlcCer to the cell surface upon growth in conditions of high CO_2_ and neutral/alkaline pH could be explained by biological changes due to an increased production or/and to an increased trafficking of GlcCer. To discern between the two possibilities, several scenarios should be considered. For one, increased expression of *GCS1* during these conditions could explain this increased surface GlcCer localization. To test this, reverse transcription PCR was performed on cells grown in the permissive and the restrictive conditions using primers specific to the cryptococcal *GCS1* gene. The forward and reverse primers bind different exons, so that the transcript from cDNA template would be distinct in size from the transcript of any contaminating genomic DNA (gDNA). There were no apparent differences in the amount of transcript seen using this technique from *Cn* grown at neutral/alkaline compared to those from acidic pH (Figure S1).

**Figure 5:**
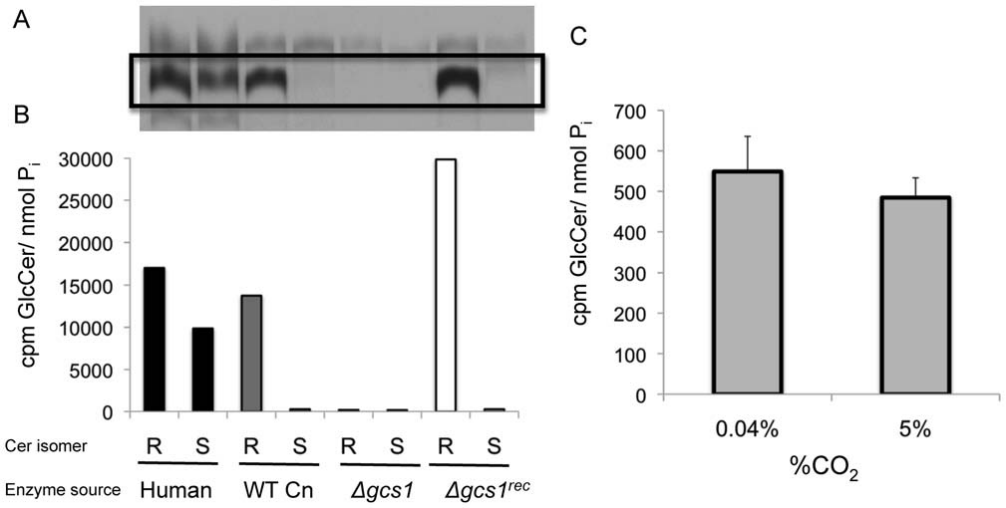
*In vitro* assay of *GCS* activity in *Cn*. An *in vitro* assay for *GCS* activity in *Cn* was successfully adapted from previously described methods. (A.) The bands from the thin layer chromatography corresponding to GlcCer (box) were quantified using a scintillation counter. The radioactive signal was normalized to the total amount of protein (B). The activity of human *GCS*, wild type (WT) Cn, *Δgcs1*, and *Δgcs1-*reconstituted strains was assessed using both the R and S isomers of the ceramide substrate. (C.) *In vitro* activity of WT Cn was evaluated at pH 7.2 and either high (5%) or low (0.04%) CO_2_.

**Figure 6:**
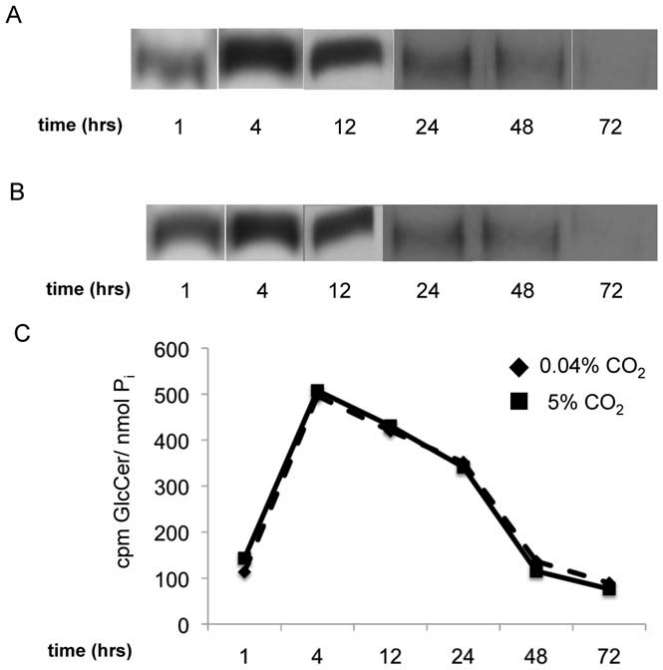
*In vivo* production of GlcCer in *Cn* grown in high and low CO_2_. To determine if *in vivo* GlcCer production is affected by CO_2_ concentration as seen in the Δgcs1 strain, wild type Cn was grown at various time points (below), then given radiolabeled ^3^H-palmitate for two hours. This *in vivo* assay shows the incorporation of ^3^H-palmitate into more complex lipids, such as GlcCer. The lipids were extracted from Cn grown at 0.04% (A) and 5% (B) CO_2_. Lipids were separated on TLC before being exposed to film to visualize. The production was tested at time points from 1–72 hours in the growth curve of Cn. These bands were quantified for the amount of radioactive signal using a scintillation counter, and normalized to inorganic phosphate (P_i_) as a measure of cell number (C).

**Figure 7:**
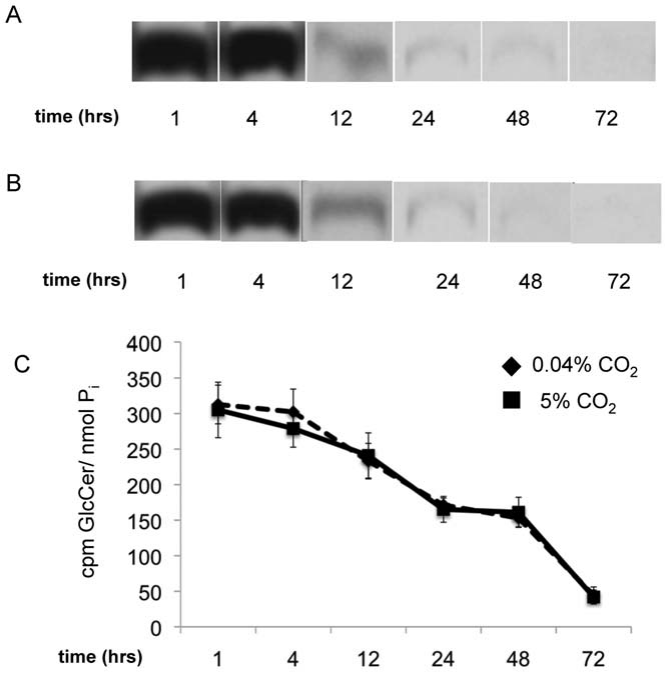
*In vivo d*egradation of GlcCer in *Cn* grown at high or low CO_2_. To determine if these growth conditions affect the degradation of GlcCer *in vivo,* a pulse of radiolabeled palmitate was given to live cultures at either high or low CO_2_. Cells were collected at set time points after the palmitate was removed and the amount of GlcCer was analyzed on film from 1–72 hrs at 0.04% CO_2_ (A) and 5% CO_2_ (B), then quantified with a scintillation counter (C). GlcCer showed little or no degradation in either condition during the selected time frames.

Another possibility is that the Gcs1 enzyme itself has a higher activity rate when grown at the more neutral/alkaline pH of 7.2–7.4. This must be tested for both *in vitro* activity and *in vivo* production. *In vitro* activity assays for GlcCer synthase in fungi have been reported [27], however these techniques did not work for *Cn* in our hands, and had to be adapted for these purposes. Using the technique described here, the *in vitro* activity of Gcs1 in *Cn* was determined using different substrates (Figure 5A and 5B). Ceramide species used as substrates in this assay were either R or S isomers, with respect to the α-OH group on the acyl chain. Membranes containing human GCS enzyme and wild type *Cn* Gcs1, as well as membranes from the reconstituted *Cn* strain (*Δgcs1* ^REC^) and the *Δgcs1* were tested for *in vitro* Gcs1 activity. While the human enzyme appears to be able to use both R- (the natural conformation) and the S- (a synthetic conformation) isomers of ceramide as a substrate, the Gcs1 from *Cn* showed substrate specificity for the naturally occurring isomer. The membranes from the *Δgcs1* strain showed no activity. Once established, this assay was used to determine if differences in enzyme activity exist between membranes from *Cn* grown at pH 7.2 in either high (5%) or low (0.04%) CO_2_ (Figure 5C). Note that whereas the CO_2_ concentration was varied in this experiment, the pH chosen (7.2) still allowed this to be a comparison of conditions which are prohibitive (pH 7.2, high CO_2_) and permissive (pH 7.2, low CO_2_) to *Δgcs1* growth. This was done for this assay alone because *in vitro* assays are sensitive to pH changes. After quantification of radiolabeled GlcCer, the *in vitro* activity of Gcs1 showed no significant differences when cells were grown at either high or low CO_2_. These *in vitro* examinations of Gcs1 activity do not provide information with regards to the production of GlcCer in living *Cn* cells. To assess this parameter fully, an *in vivo* assay of GlcCer production was performed by adding a radiolabelled palmitate, which will be incorporated in ceramide and, thus in GlcCer. At several time points ranging from short term to stationary phase of growth, production of GlcCer was determined in *Cn* grown in minimal media at 5% CO_2_ in either neutral (7.2) or acidic (4.0) pH. Across all time points examined, there was no significant difference in *in vivo* production of GlcCer when grown at these time points (Figure 6). GlcCer degradation was also examined in these conditions using a similar assay with a pulse-chase experimental design. This degradation rate showed no differences in cells grown at pH 4.0 compared to pH 7.2 (Figure 7).

These examinations of *in vivo* production of GlcCer in culture only take into account the amount produced during specific time points tested, but it does not provide information on the total mass of GlcCer. To address this, the total lipids were extracted from cultures of wild type (Figure S2A) and *Δcap59* (Figure S2B) grown at various time points at 5% CO_2_ in either pH 4.0 or 7.2. These lipids were subjected to mass spectroscopy to quantify the amount of fungal GlcCer, normalized to cell number. There were no significant differences in the total amount of GlcCer from *Cn* grown at pH 4.0 compared to pH 7.2 at any of the time points examined. Taken together, these studies suggest that the differences in surface localization of GlcCer during infection and growth at infection-like *in vitro* conditions may be due to and increased trafficking of GlcCer to the surface and not to an increased synthesis. Please note that intracellular cytosolic GlcCer is not visualized with our methods because cells are not permeabilized with Triton X and, thus, IgM F09 primary does not enter the cell. This fixation-based method was preferred to avoid a possible mis-localization or redistribution of GlcCer from the surface during the experiment.

### Cz hydrolyzes fungal GlcCer *in vitro*

As mentioned above, the ultimate goal for studying pathways crucial to virulence of fungi like *Cn* is to develop therapeutic strategies that target these pathways. The phenotype of the *Δgcs1* strain shows a critical role for the production of GlcCer in *Cn* virulence. While targeting the fungal Gcs1 enzyme has, to date, proven unsuccessful with existing inhibitors in *Cn* (unpublished Del Poeta lab data), we hypothesize that targeting GlcCer directly would be an effective way to recapitulate the *Δgcs1* avirulent phenotype in wild type *Cn* during infection. Previous experiments here showing that GlcCer is localized to the cell surface during conditions where the *Δgcs1* strain shows growth defects further suggest that targeting the GlcCer directly could have therapeutic significance.

**Figure 8:**
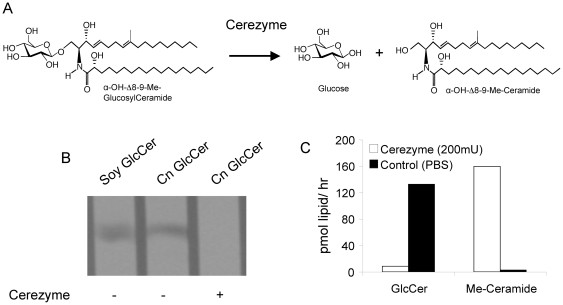
Treatment of fungal GlcCer *in vitro* with Cerezyme. A) Enzymatic reaction leading to the degradation of GlcCer in *Cn* catalyzed by beta-glucosidase (Cerezyme). B) Extracted cryptococcal GlcCer (8 µg) was treated *in vitro* with 200 mU/ml of Cerezyme for 1 hour at 37°C. The reaction was stopped, lipids extracted, and loaded on a TLC with a soy standard for GlcCer. The GlcCer was visualized with a resorcinol spray. C) The same amount of GlcCer was treated with 200 mU/ml of Cerezyme and the amount of GlcCer was analyzed with mass spectrometry, along with the amount of the reaction product, α-OH-Δ8-Me-Ceramide (Me-Ceramide). The GlcCer treated with Cerezyme showed complete hydrolysis in these conditions.

There are considerations to address before examining the efficacy of targeting GlcCer in *Cn*. Structural differences exist between the GlcCer species found in humans and in fungi [19], [28]. Notable differences in the structure of fungal GlcCer include the methylation at position 9 of the sphingoid backbone (Figure 8A), compared to the human GlcCer (which does not have this modification). To determine if Cz can effectively hydrolyze fungal GlcCer *in vitro,* 8 µg of purified *Cn* GlcCer were incubated with 200 mU/mL of Cz for 1 hour at 37°C, then visualized on TLC (Figure 8B) and later measured with mass spectroscopy (Figure 8C). Treatment with Cz showed a significant decrease in the amount of *Cn* GlcCer with a concomitant increase in 9-methyl-ceramide (precursor to GlcCer synthesis), whereas PBS-treated showed no hydrolysis (Figure 8B and 8C).

### Cz hydrolyzes fungal GlcCer in growing cultures of *Cn*

**Figure 9:**
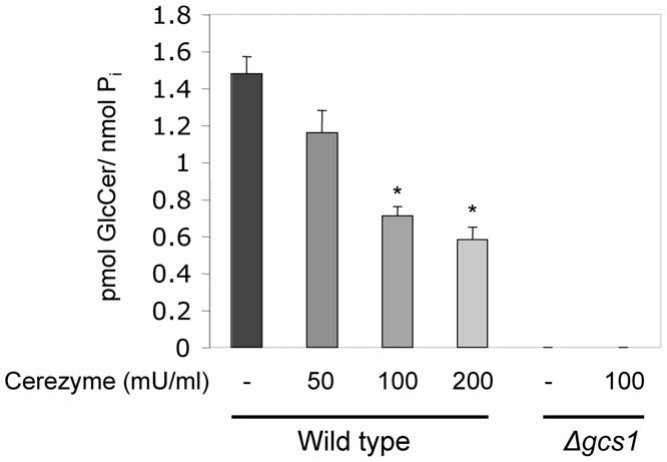
Cerezyme treatment effect on the amount of GlcCer in cultures of *Cn.* Mass spectroscopy was used to analyze amount of GlcCer of wild-type H99 or *Δgcs1* mutant strain treated with different concentrations of Cerezyme for 1 hour at 37°C normalized to phosphate (P_i_). Enzyme treatment hydrolyzed GlcCer in a dose-dependent manner. *, *P<*0.05 by Student *t* test, WT 100 or 200 versus untreated (−).

Many features of *Cn* biology not seen in the previous *in vitro* assay, including the polysaccharide capsule, could prove to be a barrier to Cz's use as a treatment. In order to potentially be used as a therapy, it must be shown that Cz treatment is also able to reduce the amount of GlcCer in growing cultures. Cultures of wild type *Cn* and the Δ*gcs1* strain were treated with increasing amounts of Cz for 1 hour at 37°C. The lipids were extracted and analyzed for GlcCer content by mass spectroscopy (Figure 9). Cz treatment of wild type *Cn* showed a dose-dependent decrease in GlcCer up to 200 mU/mL. Δ*gcs1* had no detectable GlcCer with or without Cz treatment.

### Cz treatment induces membrane integrity defects in wild type *Cn* cells *in vitro*

**Figure 10:**
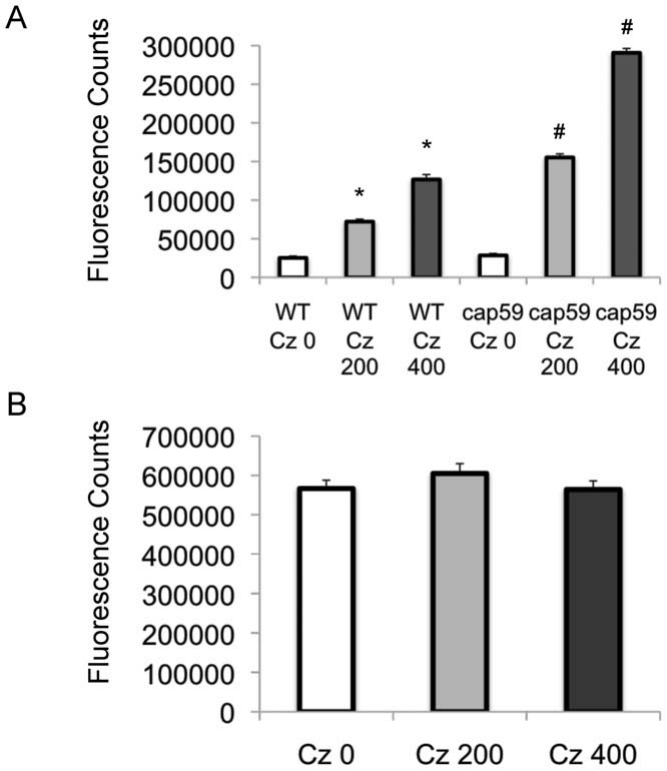
Cerezyme treatment reduces membrane integrity of *Cn.* Membrane integrity is indirectly measured using a dye, SYTOX, that fluoresces upon entering a cell, but is excluded from functional membranes. This is measured in arbitrary units of fluorescence (with background subtracted). The strains tested here were WT Cn and *Δcap59* (A) and *Δgcs1* (B). WT and *Δ*cap59 strains show increasing fluorescence when treated with Cerezyme (0, 200 mU/ml, or 400 mU/ml), but *Δgcs1* remains unaffected by treatment. *Δgcs1* also shows a much higher signal than other strains. *, *P<*0.05 by Student *t* test, WT 200 mU/ml or 400 mU/ml versus untreated; #, *P<*0.05 by Student *t* test, acapsular 200 mU/ml or 400 mU/ml versus untreated.

Based on Δ*gcs1* phenotype (hypersensitivity to SDS), a potential cause for the lack of viability in this strain is a defect in membrane integrity. If Cz treatment recapitulates the Δ*gcs1* phenotype, as hypothesized, treatment with this enzyme should induce a similar membrane integrity defect. To test this, we used the fluorescent dye SYTOX, which is excluded from functional membranes. When SYTOX enters the cell, it can bind to nucleic acids, causing it to fluoresce. Here we examine two dosages of Cz compared to vehicle control on the WT *Cn,* Δ*cap59* (Figure 10A), and Δ*gcs1* strains (Figure 10B). Notice that the Δ*gcs1* strain shows a high fluorescent signal, indicating a defect in its ability to exclude SYTOX from the cell. While the magnitude is much lower, the treatment with Cz induces a dose-dependent increase in SYTOX signal in both WT *Cn* and Δ*cap59* strain.

### Cz treatment reduces *in vitro* growth of *Cn* in high but not low CO_2_

**Figure 11:**
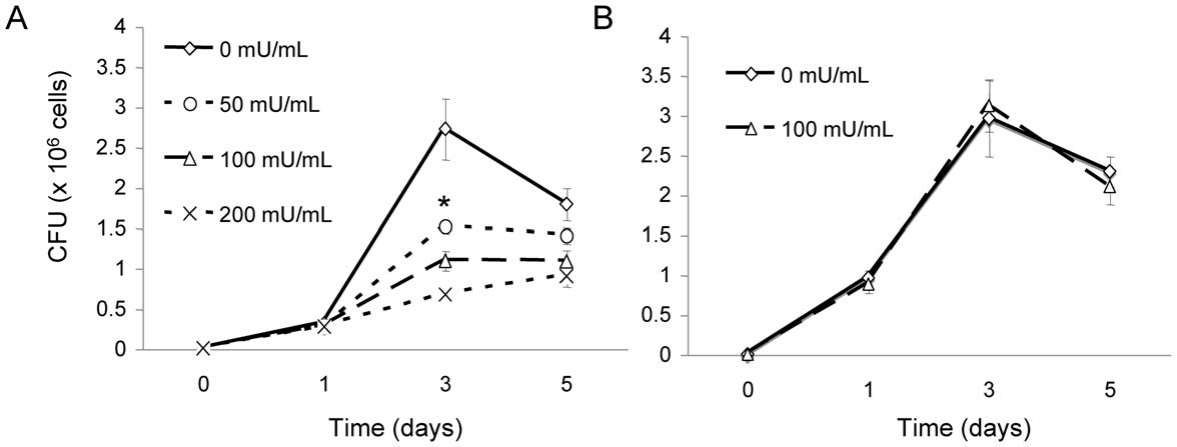
Cerezyme treatment effect on the *in vitro* growth of *Cn.* I*n vitro* growth of *Cn* was assessed in minimal media, pH 7.2, at either 5% (A) or 0.04% (B) CO_2_ with different concentrations of Cerezyme (0, 50, 100 or 200 U/mL). Treatment shows a dose-dependent inhibition of fungal growth. This growth reduction was not observed in the cells grown at low (0.04%) CO_2_. *, *P<*0.05 by Student *t* test, WT 50 mU/ml or 100 mU/ml or 200 mU/ml versus untreated.

Now that Cz has proven to be effective in hydrolysis of fungal GlcCer, the effect of Cz on the growth of *Cn* was determined. In minimal media, 10^4^ *Cn* cells were grown at pH 7.2 in either high (Figure 11A) or low CO_2_ (Figure 11B). These conditions were chosen because they were restrictive and permissive to the growth of Δ*gcs1*, respectively. Concentration of Cz ranged from zero (no treatment) to 200 mU/mL. Figure 11 illustrates how concentrations of Cz reduced the growth of *Cn in vitro* compared to no treatment but only at 5% CO_2_, and not at 0.04% CO_2_.

### Cz hydrolyzes fungal GlcCer from *Cn* during infection in mouse models

**Figure 12:**
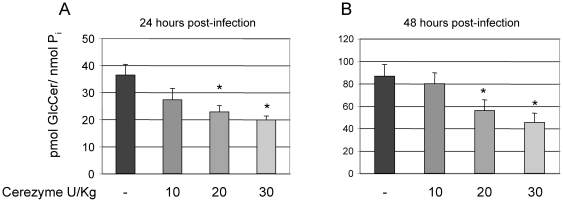
Cerezyme treatment decreases fungal GlcCer in mouse models of *Cn* infection. Mass spectrometry analysis of GlcCer of *Cn* recovered from the lung of infected mice for 24 (A) or 48 (B) hours. Enzyme treatment hydrolyzed GlcCer in a dose-dependent manner. *, *P<*0.05 by Student *t* test, 24 or 48 hours 20 U/Kg or 30 U/Kg of Cerezyme versus untreated.

For potential translation of this work into infection models, the ability of Cz to hydrolyze GlcCer from *Cn* in the mouse lung was assessed. Thus, mice were infected with 10^7^ *Cn* cells intranasally. Immediately after the inoculation of *Cn* cells, mice were given Cz intranasally (either 10, 20, or 30 U/kg, or vehicle alone). Though the dosage recommendations for Cz in humans varies, these dosages used are within the parameters of those used in human therapy. Mice were sacrificed at either 24 (Figure 12A) or 48 hours (Figure 12B) and the fungal cells were removed from the lung by broncheoalveolar lavage (BAL). After separation from alveolar macrophages, the lipids were extracted from *Cn* cells and analyzed by mass spectroscopy for fungal GlcCer content. Cz treatment showed a dose-dependent decrease in this model as well, in both 24 and 48-hour time points.

### Cz treatment significantly prolongs the life of mice in an inhalation murine model of cryptococcosis

**Figure 13:**
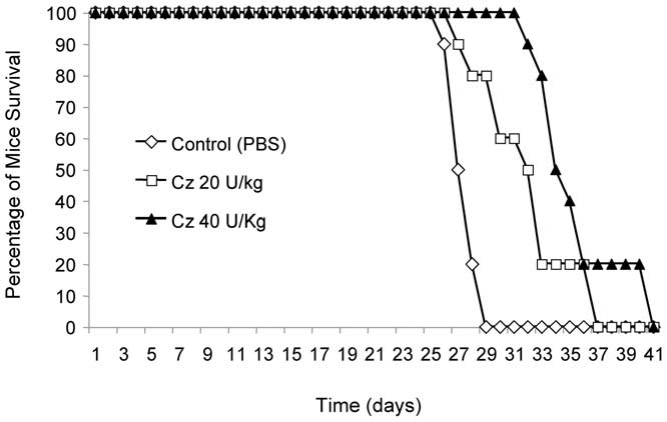
Cerezyme treatment in mouse models of infection with *Cn.* CBA/J mice (n = 10 per group) were infected with 5×10^5^ *Cn* wild type H99 strain intranasally. Mice were treated with PBS, 20 U/kg or 40 U/kg of Cerezyme (Cz) immediately after the injection of fungal cells and every 2 days. The higher dose of Cz conveyed a protective effect to infected mice, showing a significant increase in survival (by Wilcoxon-Rank Sum test *P*<0.01, compared to PBS treated mice). The data are representative of two separate experiments.

Finally, the effect of Cz on the virulence of *Cn* was evaluated. We used the inhalation murine model of *Cn* infection that is both widely used and clinically relevant. This model was also chosen based on the phenotype of the *Δgcs1* strain, which did not cause meningoencephalitis when administered intranasally, highlighting the fact that the role of GlcCer occurs in the lung infection. Thus, mice were infected with 5×10^5^ *Cn* cells and treated 20 minutes later with either vehicle (PBS), 20 U/kg, or 40 U/kg of Cz, intranasally. These treatments were repeated every other day until death. Their survival was monitored (Figure 13). While both treatment groups showed a benefit with the administration of Cz, the higher dose showed a highly significant increase in the lifespan of the mice. The average post-infection day of death was 27.6 days +/− 0.97 for vehicle alone (PBS), 31.4 days +/− 3.4 for 20 U/kg of Cz, and 35.5 days +/− 2.4 for 40 U/kg of Cz. The 40 U/kg dose showed significant increase in survival as determined by the Wilcoxon Rank Sum Test (p<0.01) compared to vehicle alone.

## Discussion

Previous studies have shown that glucosylceramide synthase is essential for the pathogenicity of *Cn* [18]. Targeting this system, therefore, has potential use in novel antifungal therapies. Past attempts to use existing inhibitors of mammalian GlcCer synthase revealed that they do not reduce the activity of the fungal enzyme or affect the growth of *Cn*. However, studies targeting fungal GlcCer using antibodies against it have been shown to have anti-cryptococcal affects *in vitro* [22] and confer passive immunity to mice infected with *Cn* [26]. In this study, we exploited fungal GlcCer as a potential target.

First, we studied the localization of GlcCer and its production during infection-like conditions and the effects of the enzymatic degradation of fungal GlcCer on cryptococcal growth and infection. Using immunofluorescence, we showed an increased localization of GlcCer to the cell surface during infection. GlcCer has been shown to be trafficked through the cell membrane, cell wall, and into the extracellular environment in vesicles, so it is possible that GlcCer could be found in any of these structures. Next, we determined that this increase is a reflection of increased trafficking to the membrane by excluding changes in Gcs1 expression or activity, GlcCer production or degradation, and total GlcCer during growth at the conditions that mimic the extracellular spaces of the lung environment. While many other molecules in *Cn* may show differential trafficking during growth at these conditions, GlcCer is of particular interest due to the clinically relevant phenotype of conditional avirulence in the *Δgcs1* mutant.

Despite this exclusionary data being negative per se, there are still a few pieces of information about the biology and biochemistry of *Cn* learned from these experiments. We adapted a reliable biochemical assay for *in vitro* Gcs1 activity in *Cn,* which showed not only a substrate specificity on the stereochemistry of the α-OH group of the ceramide acyl chain, but can also be a surrogate measurement for amount of Gcs1 at the membrane. Additionally, whereas these studies showed no differences in GlcCer production or degradation in living cultures of *Cn,* we show here that palmitate radiolabeling is a reliable method for examining lipid profiles of growing fungal cultures at various points in the growth curve, and can also be used to examine lipid degradation. While the specific localization (e.g. cell wall, capsule) cannot be accurately assessed with the resolution provided by the immunofluorescence alone, the increased localization to the surface during infection makes the *Cn* GlcCer more vulnerable to external enzymatic degradation.

To exploit this hypothesis, we used the recombinant human enzyme, Cz, which hydrolyzes the glucose moiety of mammalian GlcCer. We found that Cz degrades fungal GlcCer *in vitro*, in cultures of *Cn*, and in *Cn* that reside in the mouse lung. These experiments were needed in order to show the feasibility of using a human enzyme on the similar yet distinct fungal GlcCer and also to assess the plausibility of this enzyme being used in the context of a mammalian host lung. Recall that human inhibitors of GlcCer synthase do not affect the cryptococcal enzyme Gcs1. In terms of substrate specificity, the human enzyme has been shown to glycosylate many different substrates, while the cryptococcal enzyme has a more narrow range of specificity [15]. Though Cz (human glucocerebrosidase) is a distinct enzyme from human GlcCer synthase, it is reasonable to assume that the catabolic human enzyme, Cz, would also have to recognize multiple substrates.

In the experiments from Figures 9 and 12 where actual *Cn* cells are used, there seems to be a plateau in the ability of Cz to hydrolyze GlcCer. One explanation for this is that these experiments were conducted at the physiological pH found in the extracellular spaces of the lung (7.2–7.4), whereas the optimum pH for Cz activity is more acidic. This could have limited the enzymatic activity of Cz at a protein level. Another possibility is that *Cn* has a well-studied polysaccharide capsule that can act as a barrier. This may account for the plateau in hydrolysis seen. Finally, *Cn* is known to secrete several proteases [29]. These enzymes may inactivate Cz to a point where increased concentration will not correlate to increased activity.

To characterize the effects of Cz treatment on *Cn* biology, we used the dye SYTOX, which fluoresces upon binding to nucleic acids, but is excluded from functional membranes. We showed that the membrane from the *Δgcs1* strain has an impaired ability to exclude SYTOX, indicating a membrane integrity deficit in this strain. This is supported by the observation that the *Δgcs1* has a growth defect in media supplemented with SDS [18]. Indeed, upon treatment with increasing concentrations of Cz, a dose-dependent increase in membrane permeability to SYTOX in both wild type *Cn* and *Δcap59* strains was observed. Even at the maximum dose examined, however, the degree of SYTOX signal from *Δgcs1* strain was higher than the Cz-treated strains (compare CZ 0 in Figure 10B with WT Cz 400 in Figure 10A). This was expected as Cz treatment does not hydrolyze all GlcCer, whereas the *Δgcs1* cells have no GlcCer. Of note, the *Δcap59* strain showed a baseline reduction in the ability to exclude SYTOX when compared to the wild type *Cn.* The capsule connection to the cell wall may confer some additional stability to the cell surface in the wild type that is not seen in the acapsular strain. Taken together, these experiments shoed that Cz treatment of wild type *Cn* recapitulates membrane deficits found in *Δgcs1* strain.

Based on previous experiments reported here and the avirulent phenotype of the *Δgcs1* strain, we hypothesized that removal of GlcCer by exogenous treatment with Cz would induce similar deficits in wild type *Cn,* both *in vitro* and during infection. The *in vitro* effects of Cz on *Cn* growth were then assessed. As shown, Cz treatment reduced the growth of *Cn* at high CO_2_ but not at low CO_2_, most notably at 48 hours of growth. At 24 hours of growth, there were little differences in the treated and untreated groups during incubation at high CO_2_. This is not entirely unexpected, as the growth defects in the *Δgcs1* strain itself (with no GlcCer at all) only first start to appear at 24 hours of growth. This finding is significant, as Cz treatment should theoretically recapitulate the *Δgcs1* phenotype, as shown above with membrane integrity experiments. This pattern of growth inhibition with Cz treatment is consistent with the *Δgcs1* phenotype, which shows growth differences compared to wild type *Cn* at high but not low CO_2_.

We hypothesized that treatment of infected mice would reduce the growth of *Cn* in the extracellular spaces of the lung, and prolong host survival. Infected mice were treated with two different doses of Cz, and survival was compared to vehicle control. The higher dose of Cz showed significant increase in length of survival time in the treated mice. We further speculate that the Cz-mediated reduction in extracellular growth in the lung allowed the mouse immune system to delay the eventual dissemination of *Cn*, increasing the time to mortality. To assess this, additional experiments are needed to examine the physio-pathology of the lung infection in treated and untreated animals. Cz treatment did not prove curative or completely protective against the infection, as all mice tested eventually died. This could be due to the fact that the optimal activity of Cz is at more acidic pH than that found in the extracellular spaces of the lung, or that Cz tends to accumulate in the phagolysosomes, where GlcCer hydrolysis would have no effect (even *Δgcs1* can grow at pH 4.0). Another potential for Cz use is as an adjunct therapy in instances of resistance or unmanageable side effects with standard antifungal drugs (e.g. fluconazole). This hypothesis is supported by our preliminary investigations in which the combination of Cz and fluconazole has a strong synergistic effect against *Cn in vitro* and by recent studies in *C. albican* in which alteration of GlcCer synthesis renders the fungus hypersensitive to fluconazole [30].

**Figure 14:**
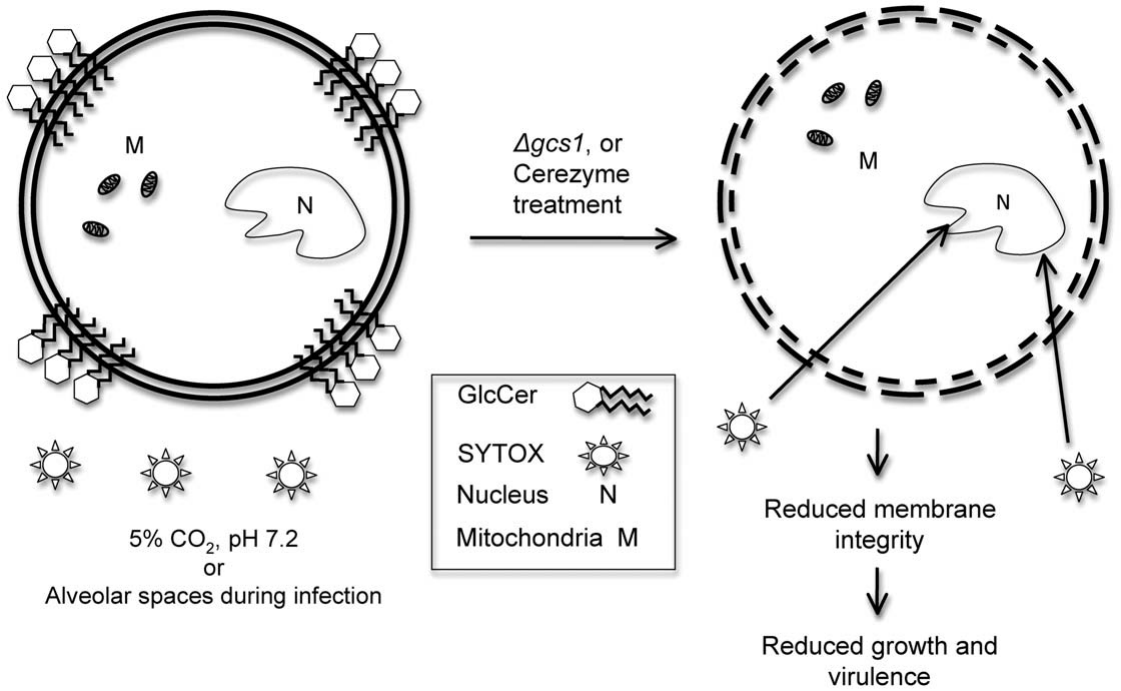
Schematic of GlcCer localization during infection and the proposed effect of GlcCer reduction in Cerezyme treatment or *Δgcs1* strain.

The major findings of this study are illustrated in a schematic form in Figure 14. This study showed that targeting the GlcCer system in general has clinical significance. Though currently no fungal Gcs1 inhibitors exist, targeting the enzyme directly in a fungus-specific manner would be theoretically effective, and this study provides justification for the development of such compounds. Also, further dissecting the role of enzymes upstream in the sphingolipid pathway leading to GlcCer synthesis could yield even more therapeutic targets based on the same rationale used to study Cz here.

## Materials and Methods

### Ethics Statement

This study was carried out in strict accordance with the recommendations in the Guide for the Care and Use of Laboratory Animals of the National Institutes of Health. The protocol was approved by the Medical University of South Carolina Institutional Animal Care and Use Committee (Permit Number: 2019). All animal procedures were performed according to the approved protocol, and all efforts were made to minimize suffering.

### Strains and media

The strains used in this study were wild type *Cryptococcus neoformans* (*Cn*) H99 strain and the *Δgcs1* mutant, which was created in our laboratory [18]. *Saccharomyces cerevisiae* strains expressing human GCS under a galactose-inducible promoter were used for the *in vitro* enzyme activity of the human enzyme and were created previously in our lab [18]. All strains were grown in YNB (yeast nitrogen broth, Sigma-Aldrich) with 2% glucose and 50 mM HEPES as a buffer, at either pH 4.0 or 7.2, as indicated. All strains were grown at 37°C for all assays reported.

### *In vitro* GCS assay

The *in vitro* GCS assay reported here was adapted from a previous GCS assay in the fungus *Pichia pastoris* [27]. Briefly, this assay used bead-disruption to lyse *Cn* cells and ultracentrifugation (100,000×g for 30 min) to separate the lipid/membrane fraction from the rest of the cell components. This membrane fraction is used as the source of GCS enzyme in this assay, as purified fungal GCS is not yet available. The 0.75 mg of membrane fraction is suspended in reaction buffer (100 mM Tris/HCl, pH 8.0, 15% glycerol). Radiolabeled UDP-Glucose (C^14^) was purchased from American Radiolabeled Chemicals and 500,000 dpm was added (10 GBq/mmol, final concentration 8 µM). To this, R-α-OH-C16-ceramide from MUSC Lipidomics Core was added for a final concentration of 0.3 mM. Triton X 100 was added for a final percentage of 0.5% in a final volume of 100 µL. The mixture was vortexed and sonicated for 30 second cycles, then incubated at 37°C for 45 minutes. At the end, 0.9 mL of 0.45% NaCl solution was added, as well as 4 mL of chloroform:methanol 2∶1 to stop the reaction and achieve phase separation. A portion (1/4) of the extracted lipids from the lower, organic phase of the extraction were set aside for inorganic phosphate determination (P_i_). Lipids were then dried down in an SPD 2010 Speedvac (Thermo Electron Corp.) and suspended in 50 µL of chloroform:methanol 2∶1. Samples were then run on a thin layer chromatography (TLC) plate along with a soy GlcCer standard for identification. The tank solvent system used was chloroform: methanol: water in the ratio 97.5∶ 7.5∶6. Sugars were identified by using a resorcinol spray and the plate was exposed to radiosensitive film for 2 days at −80°C. Quantification was achieved by scraping the bands and running samples in an LS 6500 scintillation counter (Beckman Coulter). Values were normalized to P_i_ values.

### *In vivo* GlcCer production and degradation assay

For the *in vivo* production assay, *Cn* cells were grown in the media/growth conditions and time points. Radiolabeled palmitate (2 µCi/mL) from American Radiolabeled Chemicals was added to the media and cells. After 2 hours of incubation with the radiolabeled palmitate, the cells were pelleted and the lipids were extracted directly using the methods described below. After the Mandala extraction protocol (described below), base hydrolysis was performed on the lipids to remove most glycerolipids. Briefly, lipids were suspended in 1 mL of chloroform and incubated with 0.5 mM sodium methoxide (in methanol) for 1 hour at 45°C. After this, samples were centrifuged for 5 minutes at 3,000 rpm to achieve phase separation. The lipid extraction, drying, and TLC analysis was performed as above.

For the degradation assay, a pulse of radiolabeled palmitate was added to the culture and incubated for 2 hours. Cells were centrifuged and washed 5 times to remove external radiolabeled palmitate. Cells were then suspended and allowed to grow until the desired time points, then the lipids were extracted as described above.

### Production of IgM monoclonal antibodies against fungal GlcCer

Anti-GlcCer IgM monoclonal antibodies (mAb) were generated as follow: three Balb/c mice were infected with 10^3^ *Cn* H99 wild-type cells. At 14, 21, and 28 days post-infection, blood was collected from the saphenous vein, serum obtained and examined for the presence of IgM against *Cn* GlcCer using an ELISA (see below). As a negative control, three mice were infected with 10^3^ *Cn Δgcs1* cells and at day 14, 21, and 28 serum was obtained and used in the ELISA. IgM antibodies against *Cn* GlcCer were detected in mice infected with *Cn* wild-type but not in mice infected with *Cn Δgcs1* (Figure S3). Therefore, at day 29, mice infected with *Cn* wild-type H99 were sacrificed, spleenocytes were isolated and fused to SP2/0 myeloma cells using polyethylene glycol 1500 (Roche Applied Science, Penzberg, Germany). The resulting cells were plated onto 96-well plates and selected with hypoxanthine-aminopterine-thymidine medium (Invitrogen, San Diego, CA). At 10 days post fusion, the supernatant of hybridoma cells was screened by ELISA against soy GlcCer obtained from Avanti Polar Lipids. Soy instead of *Cn* GlcCer was used for screening the hybridoma supernatants because of the commercial availability of the plant sphingolipid and because a previously made IgG monoclonal antibody against fungal GlcCer (MEST-2) also cross reacted against soy GlcCer [31]. Thus, we reasoned that an IgM against *Cn* GlcCer would also cross react against the soy sphingolipid. Positive clones were screened three times by limited dilutions and re-examined by ELISA. The positive clones were then amplified and stored in liquid nitrogen. The determination of the antibody isotyping was performed by using a Roche isostrip test following the method recommended by the manufacturer. We isolated two IgM clones: F09 and B11 mAb, which were examined for reactivity against purified GlcCer obtained from *Cn* cells. IgM-B11 contains a kappa light chain whereas IgM-F09 contains a lambda light chain. F09 and B11 were further purified according to previously described methods [32]. The concentration of each mAb obtained was determined using Bio-Rad protein assay.

### ELISA

ELISA was performed by coating 96 well plates (Nunc maxisorp) with 5 µg GlcCer, which was obtained as follow: soy GlcCer was from Avanti (Avanti 131304P), *Cn* and *Ca* GlcCer were purified from *Cn* wild-type strain H99 and *Ca* wild-type strain A39, respectively; mouse GlcCer was extracted from mouse peritoneal macrophage cell line J774.16. GlcCer was isolated and purified from fungi or mammalian cells following a protocol previously described [15]. Galactosylceramide (GalCer was obtained from Avanti (KRN7000). Other lipids described in Figure 1 were obtained from the MUSC Lipidomic core facility. The lipid-coated plates were dried overnight. The plates were then blocked with 5% BSA in phosphate buffered saline (PBS) for 1 hour at 37°C and then washed three times with 0.1% PBS-Tween 20. One hundred µl of supernatant containing 50 µl of mouse serum or hybridoma supernatant (diluted at 1∶24 with PBS) were added and the plates were incubated 1 hour at 37°C followed by three washes with 0.1% PBS-Tween 20. For F09 or B11, 50 µl of 1∶64 dilution of 1 mg/ml was used. Peroxidase-conjugated secondary anti-mouse IgM antibody (Sigma A786) diluted 1∶30,000 was added and the plates incubated for 1 hour at 37°C. Following 3 washes with 0.1% PBS-Tween 20, color development was observed using 3,3′,5,5′ tetramethylbenzidine substrate (TMB) (Sigma T0440). The reaction was stopped by the addition of 1N HCL and optical density was measured at 450 nm and recorded. As a negative control, the secondary antibody anti-IgM was used alone.

### Immunofluorescence

Cells were grown in the appropriate conditions described. After this, 2.5 mL 5x fixation reagent (46 mL of 0.5 M potassium phosphate and 54 mL of formaldehyde) was added to 10 mL cultures of cells. After 2 hours of shaking, the cells were centrifuged at 500xg for 5 minutes at room temperature to pellet. The cells were then suspended in 1x fixation reagent and allowed to incubate, shaking, overnight in the fixative. After this, the cells were centrifuged for 500xg for 5 minutes to pellet. The cells were then suspended in 0.5 mL of SHA buffer (1 M sorbitol, 100 mM HEPES, 50 mM sodium azide in H_2_O, final pH 7.5). After washing twice with SHA 1 mL of buffer, 10^3^ cells were suspended in 0.5 mL of WT buffer (100 mM HEPES, 0.3 M NaCl, 2 mM sodium azide, 10 g bovine serum albumin, 0.2 mL of Tween in 200 mL H_2_0 total) with 8 µg/mL of monoclonal anti-*Cn* GlcCer antibody, and incubated with shaking at room temperature overnight. Washed with 1 mL of WT buffer four times and suspended in WT buffer with FITC-conjugated goat anti-mouse IgM secondary antibody. Incubate shaking at room temperature for one hour. Wash with 1 mL of WT buffer four times and suspend in 50 µL of WT buffer. Then, 20 µL of cells were added to glass slide coated with poly-L-lysine. ProLong Gold Antifade Reagent (Invitrogen, 5 µL) was added and the slides were given a coverslip and analyzed with confocal microscopy.

### Cz treatment *in vitro*

Cerezyme was provided generously by the Genzyme Corporation. For the *in vitro* assay, Cerezyme power was reconstituted with sterile water. GlcCer extraction form *Cn* wild-type was performed as previously described [18] and 8 µg of purified sphingolipid was incubated with Cerezyme as indicated. After incubation for one hour at 37°C, the sphingolipids were extracted by addition of an equal volume of chloroform. The mixture was centrifuged and the organic layer removed, dried, and added to a thin layer chromatography (TLC) plate (Whatman). A soy GlcCer (Avanti Polar Lipids) standard was loaded to identify the sphingolipid. The plates were run in a tank with chloroform: methanol: water in the ratio 97.5∶37.5∶6. The sphingolipids were visualized with a resorcinol spray.

### Animals survival studies and broncheoalveolar lavage (BAL)

The mice used were female CBA/J (Jackson Laboratories), age 4–6 weeks. For the infection studies, mice were injected intranasally with 5×10^5^ cells. After 20 minutes, mice were treated with either PBS, 20 U/kg of Cerezyme (Cz) or 40 U/kg of Cz. This treatment was then repeated every 48 hours and survival was monitored. Broncheoalveolar lavage was performed as previously described [33]. Cells were collected, mouse macrophages were lysed by adding 0.05% SDS, and after 5 minutes the mixture was centrifuged at 1000 g for 10 minutes. The pellet (*Cn* cells) was suspended in 1 ml PBS and 100 µl was used for CFU whereas the remaining 900 µl was used for lipid extraction.

### Lipid Extraction and Analysis

Lipids were extracted as previously described [18], [34]. The Mandala extraction reagent is incubated with cells at 60°C with intermediate vortexing and sonication. After centrifugation to remove the debris, the reagent containing the lipids is dried down. The dried lipids were submitted to MUSC Lipidomics Core Facility for analysis and quantification with mass spectroscopy using the purified *Cn* GlcCer as a standard.

### SYTOX assay

The assay was performed based on the specifications given in previous publications using fungal membranes [35], [36]. Briefly, after growth in the media and treatment with Cerezyme (either 0, 200, or 400 units/mL, for one hour), 10^4^ cells were suspended in sterile PBS and transferred into a 96-well plate. SYTOX Green dye (Invitrogen) was added to each well for a final concentration of 0.2 µM. The amount of fluorescent signal per well was measured in a Wallac 1420 multilabel counter (Perkin Elmer) with an excitation frequency of 485 nm and emission frequency of 535 nm.

## Supporting Information

Figure S1 **Expression of** ***GCS1*** **transcript in** ***Cn*** **grown at high (5%) compared to low (0.04%) CO_2_.** Reverse transcription-PCR (RT-PCR) was used with primers specific to the *GCS1* gene. Differences in *GCS1* transcript level in wild type (WT) *Cn* grown at high and low CO_2_ were the indistinguishable using this technique. The positive control was *Cn* genomic DNA (gDNA) as the PCR template instead of the transcript-derived cDNA. *Δgcs1* strain was used at the negative control, and the actin expression was used as the normalization control.
(TIF)

Figure S2 **Amount of GlcCer in wild type and acapsular** ***Cn*** **grown in high CO_2_ and either acidic or neutral pH.** The total amount of GlcCer in wild type Cn (A) and an acapsular strain of Cn, cap59 (B) grown from 16–72 hours was quantified using mass spectrometry and normalized to inorganic phosphate (P_i_). Though cap59 had more GlcCer than the wild type overall, there were no significant differences in GlcCer amount grown in different pH.
(TIF)

Figure S3 **IgM response in mice infected with** ***C. neoformans*** **.** IgM anti-GlcCer antibodies are found in serum of mice infected with *Cn* wild type H99 but not in sera of mice infected with *Cn Δgcs1* mutant by Enzyme-linked immunosorbent assay (ELISA).
(TIF)
