# Supplementary figures and images for: Surface Localization of Glucosylceramide during Cryptococcus neoformans Infection Allows Targeting as a Potential Antifungal
Source: PLoS One. 2011 Jan 21;6(1):e15572. doi: 10.1371/journal.pone.0015572 (PMC3024982; doi:10.1371/journal.pone.0015572)

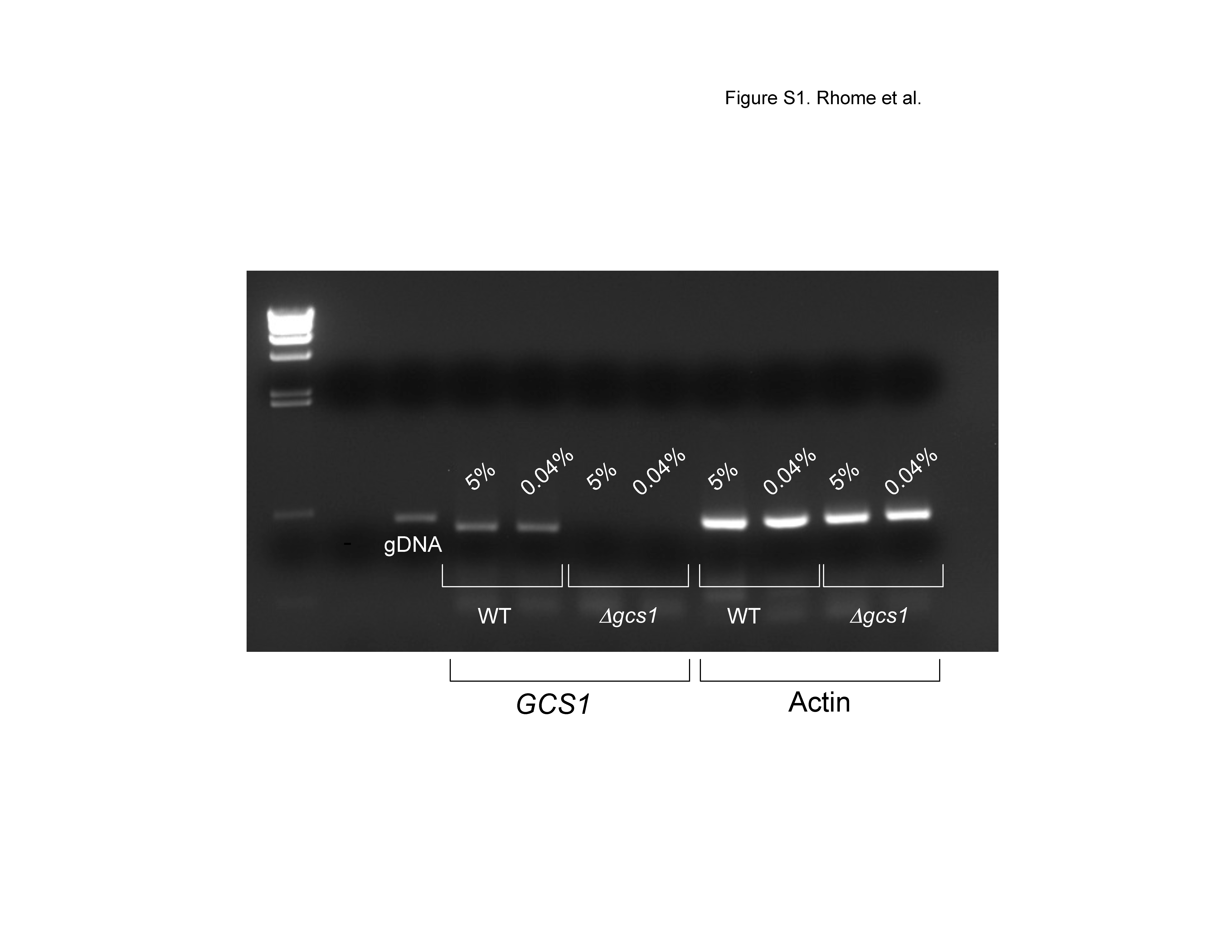

Supplement: Figure S1 — Expression of GCS1 transcript in Cn grown at high (5%) compared to low (0.04%) CO2. Reverse transcription-PCR (RT-PCR) was used with primers specific to the GCS1 gene. Differences in GCS1 transcript level in wild type (WT) Cn grown at high and low CO2 were the indistinguishable using this technique. The positive control was Cn genomic DNA (gDNA) as the PCR template instead of the transcript-derived cDNA. Δgcs1 strain was used at the negative control, and the actin expression was used as the normalization control. (TIF) [file pone.0015572.s001.tif]

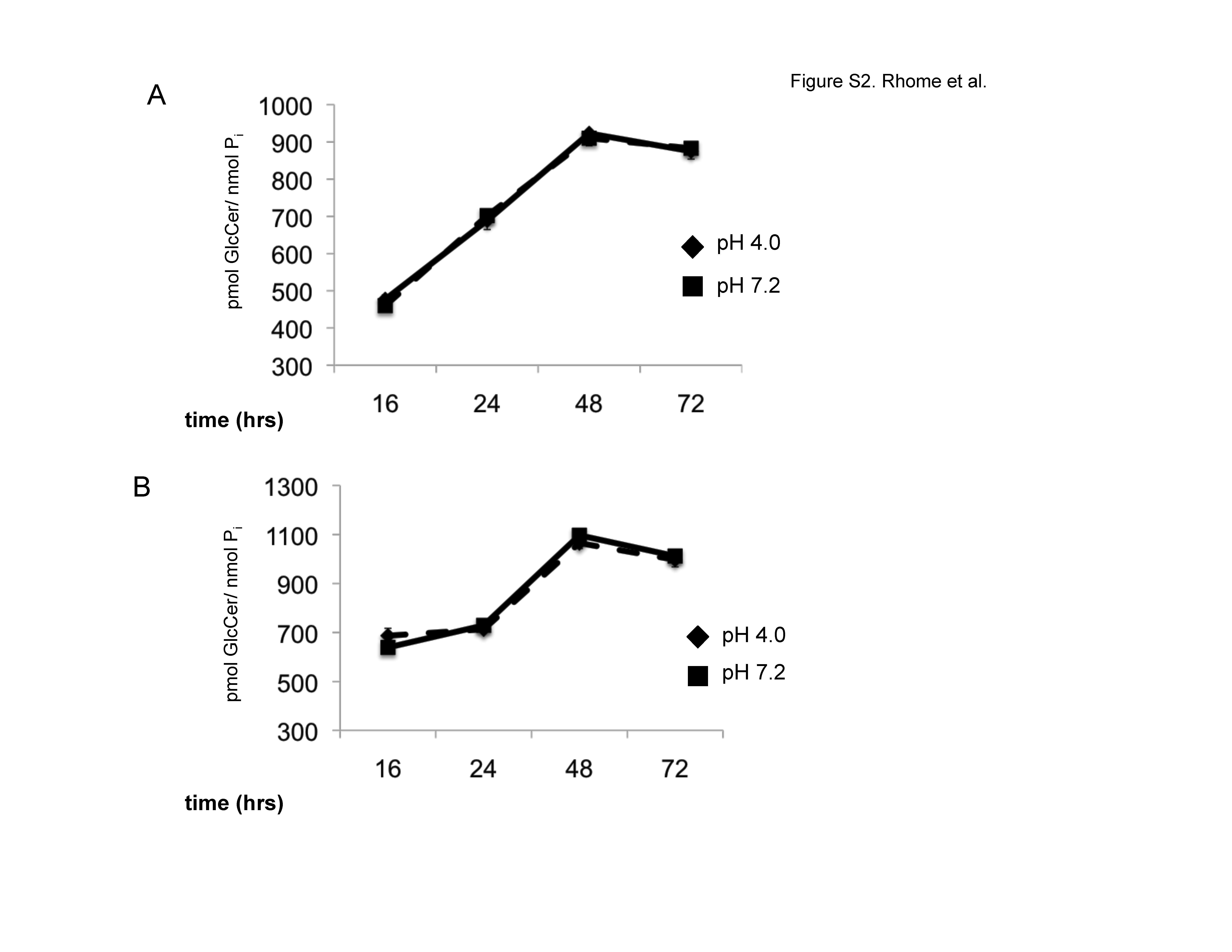

Supplement: Figure S2 — Amount of GlcCer in wild type and acapsular Cn grown in high CO2 and either acidic or neutral pH. The total amount of GlcCer in wild type Cn (A) and an acapsular strain of Cn, cap59 (B) grown from 16–72 hours was quantified using mass spectrometry and normalized to inorganic phosphate (Pi). Though cap59 had more GlcCer than the wild type overall, there were no significant differences in GlcCer amount grown in different pH. (TIF) [file pone.0015572.s002.tif]

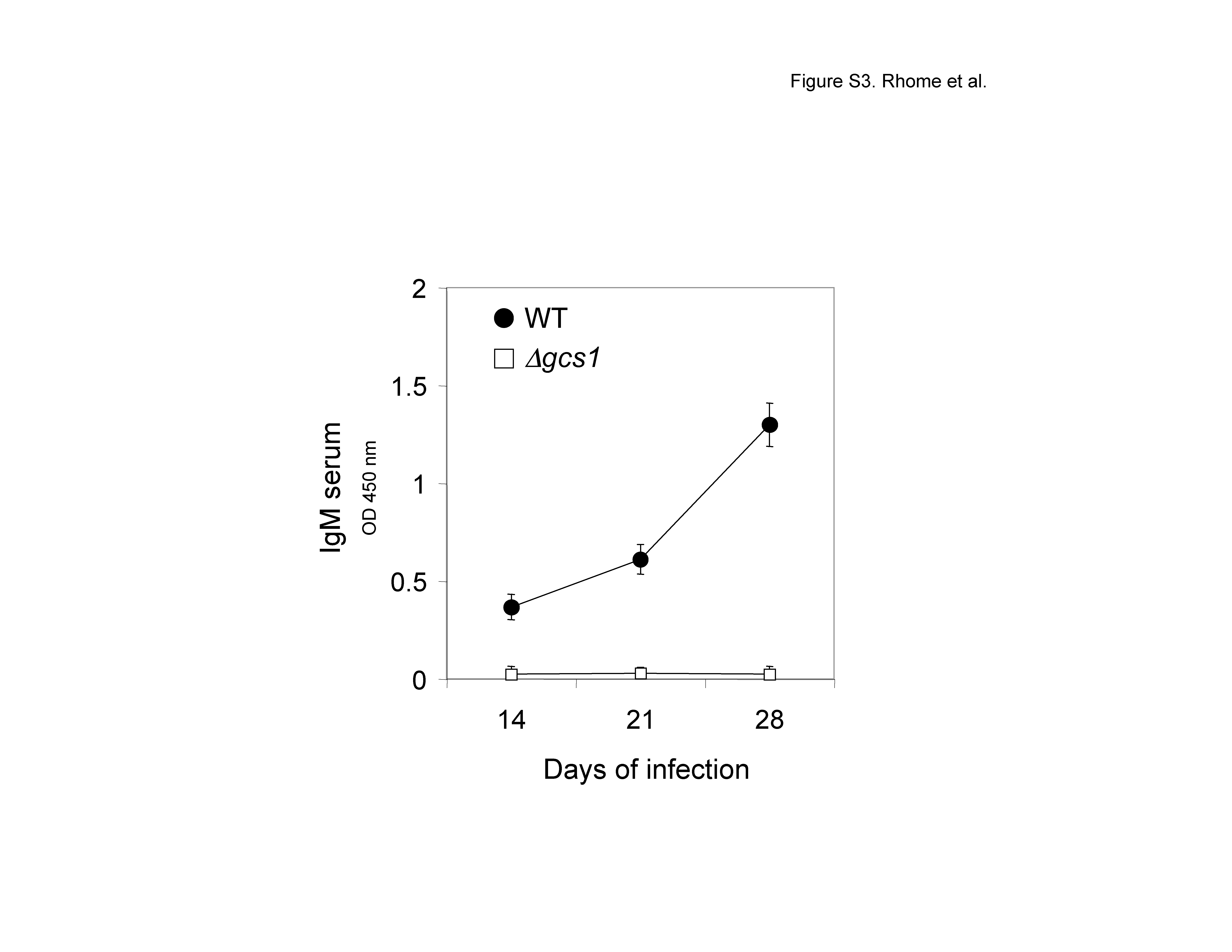

Supplement: Figure S3 — IgM response in mice infected with C. neoformans . IgM anti-GlcCer antibodies are found in serum of mice infected with Cn wild type H99 but not in sera of mice infected with Cn Δgcs1 mutant by Enzyme-linked immunosorbent assay (ELISA). (TIF) [file pone.0015572.s003.tif]
